# The Role of Functional Neuroanatomy of the Lumbar Spinal Cord in Effect of Epidural Stimulation

**DOI:** 10.3389/fnana.2017.00082

**Published:** 2017-09-22

**Authors:** Carlos A. Cuellar, Aldo A. Mendez, Riazul Islam, Jonathan S. Calvert, Peter J. Grahn, Bruce Knudsen, Tuan Pham, Kendall H. Lee, Igor A. Lavrov

**Affiliations:** ^1^Department of Neurologic Surgery, Mayo Clinic Rochester, MN, United States; ^2^Mayo Clinic Graduate School of Biomedical Sciences, Mayo Clinic Rochester, MN, United States; ^3^Department of Biological Sciences, Lehigh University Bethlehem, PA, United States; ^4^Department of Physical Medicine and Rehabilitation, Mayo Clinic Rochester, MN, United States; ^5^Department of Physiology and Biomedical Engineering, Mayo Clinic Rochester, MN, United States; ^6^Institute of Fundamental Medicine and Biology, Kazan Federal University Kazan, Russia

**Keywords:** spinal cord, swine, neuromodulation, epidural stimulation, functional neuroanatomy

## Abstract

In this study, the neuroanatomy of the swine lumbar spinal cord, particularly the spatial orientation of dorsal roots was correlated to the anatomical landmarks of the lumbar spine and to the magnitude of motor evoked potentials during epidural electrical stimulation (EES). We found that the proximity of the stimulating electrode to the dorsal roots entry zone across spinal segments was a critical factor to evoke higher peak-to-peak motor responses. Positioning the electrode close to the dorsal roots produced a significantly higher impact on motor evoked responses than rostro-caudal shift of electrode from segment to segment. Based on anatomical measurements of the lumbar spine and spinal cord, significant differences were found between L1-L4 to L5-L6 segments in terms of spinal cord gross anatomy, dorsal roots and spine landmarks. Linear regression analysis between intersegmental landmarks was performed and L2 intervertebral spinous process length was selected as the anatomical reference in order to correlate vertebral landmarks and the spinal cord structures. These findings present for the first time, the influence of spinal cord anatomy on the effects of epidural stimulation and the role of specific orientation of electrodes on the dorsal surface of the dura mater in relation to the dorsal roots. These results are critical to consider as spinal cord neuromodulation strategies continue to evolve and novel spinal interfaces translate into clinical practice.

## Introduction

Epidural electrical stimulation (EES) of the spinal cord has emerged as a promising therapy for enabling motor function (Gerasimenko et al., 2001; Minassian et al., 2004; Lavrov et al., 2008, 2015a,b; Harkema et al., 2011; Gad et al., 2013; Hachmann et al., 2013; Angeli et al., 2014; Grahn et al., 2017), respiratory muscle activation (Kowalski et al., 2013) and bladder control (Gad et al., 2016) following spinal cord injury (SCI). Several known factors can influence the effect of EES, such as spatial orientation of dorsal spinal cord structures (Holsheimer and Struijk, 1991), electrical properties of intraspinal elements (Barolat, 1998), nerve fibers activated (Holsheimer, 2002), presence of ipsi- and/or contralateral afferents (Lavrov et al., 2008) and the timing of stimulation pulses in relation to the intended motor activity (Moraud et al., 2016; Shah et al., 2016; Wenger et al., 2016). Although the dorsal roots and dorsal ascending spinal columns are considered the main target of EES (Coburn, 1985a; Rattay et al., 2000), the precise mechanisms underlying the effect of EES on these spinal neural structures remain unclear. Initial computational modeling of EES suggest that activation threshold depends on the orientation of electrical field along the target fibers (Coburn, 1985b), specifically the curvature of the dorsal root anatomy and the angles between the dorsal fibers and the spinal cord axis (Struijk et al., 1993; Holsheimer, 1998; Ladenbauer et al., 2010). More recent computer simulations further define that thick dorsal root fibers are recruited at the lowest EES intensities (Rattay et al., 2000) and particularly that Group Ia/Ib and Group II afferents are the first neural elements to be depolarized (Capogrosso et al., 2013). *In vivo* experiments in rodent models have shown that high-intensity EES leads to activation of ventral spinal neural structures that in turn produce an early response (ER) with latencies of 3–5 ms at recording sites with active muscles. At lower EES intensities, activation of dorsal spinal structures produces a middle response (MR) in muscles with latencies between 5–9 ms. The difference in timing of these two responses is likely due to an intraspinal synaptic relays from dorsal root structures to ventral horn and ventral roots (Gerasimenko et al., 2006; Lavrov et al., 2006, 2008; Courtine et al., 2009).

Computational modeling and rodent studies have shed some light on the mechanisms by which EES enables motor function after SCI; however, the role of dorsal root fiber orientation in spinal motor responses evoked by EES has not been investigated. Small animal models, such as the rodent, are not optimal for studying these structures due to significant difference in spinal cord anatomy between human and rodent spinal cord anatomy (Shah and Lavrov, 2017). Large animals including calf (Cotterill et al., 1986), sheep (Wilke et al., 1997; Kandziora et al., 2001) and swine (Bozkus et al., 2005; Navarro et al., 2012; Zurita et al., 2012; Hachmann et al., 2013; Lee et al., 2013; Guiho et al., 2017), have been successfully used as translational models. Particularly, the swine spine has gained attention as a suitable model due to its similarity to humans in terms of vertebral morphometry (McLain et al., 2002; Busscher et al., 2010; Sheng et al., 2016) and biomechanical properties (Yingling et al., 1999; Sheng et al., 2010); however, a description of the swine spinal cord anatomy and its intersegmental relationship with the spine is missing. In this study we chose the swine model due to its translational relevance (Bozkus et al., 2005; Zurita et al., 2012; Hachmann et al., 2013; Lee et al., 2013; Guiho et al., 2017; Toossi et al., 2017) and its emergence as an optimal model to study EES following SCI (Schomberg et al., 2017).

The primary goal of this investigation was to evaluate the role of spinal cord neuroanatomy in effect of EES and how spinal circuitry may inform the optimal electrode positioning in relation to spinal cord dorsal structures. More specifically, in this study we: (1) describe the gross anatomy of the swine lumbar spinal cord and the spatial orientation of dorsal spinal cord roots and rootlets (root fibers); (2) identify anatomical landmarks of the spine and spinal cord to correlate bony landmarks to spinal cord segments; and (3) determine the role of dorsal spinal cord Neuroanatomy in EES motor evoked responses.

## Materials and Methods

### Subjects

All study procedures were conducted with the approval of the Mayo Clinic Institutional Animal Care and Use Committee and in accordance with the National Institutes of Health Guidelines for Animal Research (Guide for the Care and Use of Laboratory Animals). Eleven domestic white swine males aged 8–12 weeks and weighing 25–40 kg were used for this study. Animals were kept in separate cages in a controlled environment (constant temperature at 21°C and humidity at 45%) on a 12-h light/dark cycle with *ad libitum* access to water, and were fed once daily.

### Post-Mortem Spine Dissection and Anatomical Measurements

**Figure 1:**
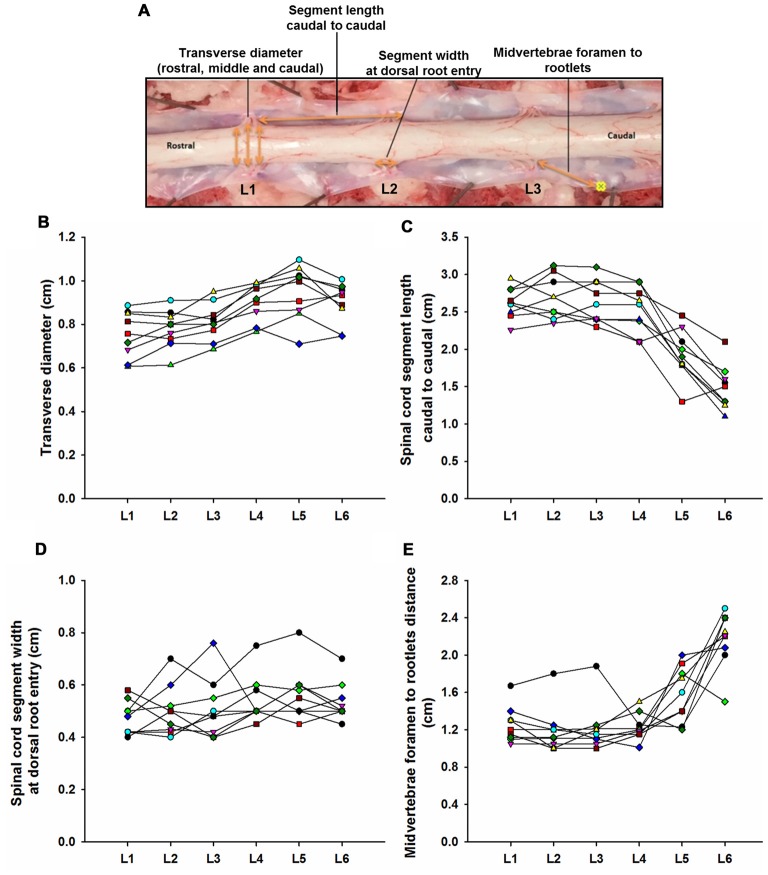
Swine’s lumbar spinal cord anatomy. **(A)** Depiction of the spinal cord anatomical landmarks identified in this study: transverse diameter, segment length caudal to caudal, segment width at dorsal root entry and midvertebrae foramen to rootlets. Data per specimen (*n* = 9) across lumbar segments is shown for **(B)** spinal cord transverse diameter, **(C)** segment length (caudal to caudal root distance), **(D)** segment width at dorsal root entry zone and **(E)** midvertebrae foramen to rootlets distance.

**Figure 2:**
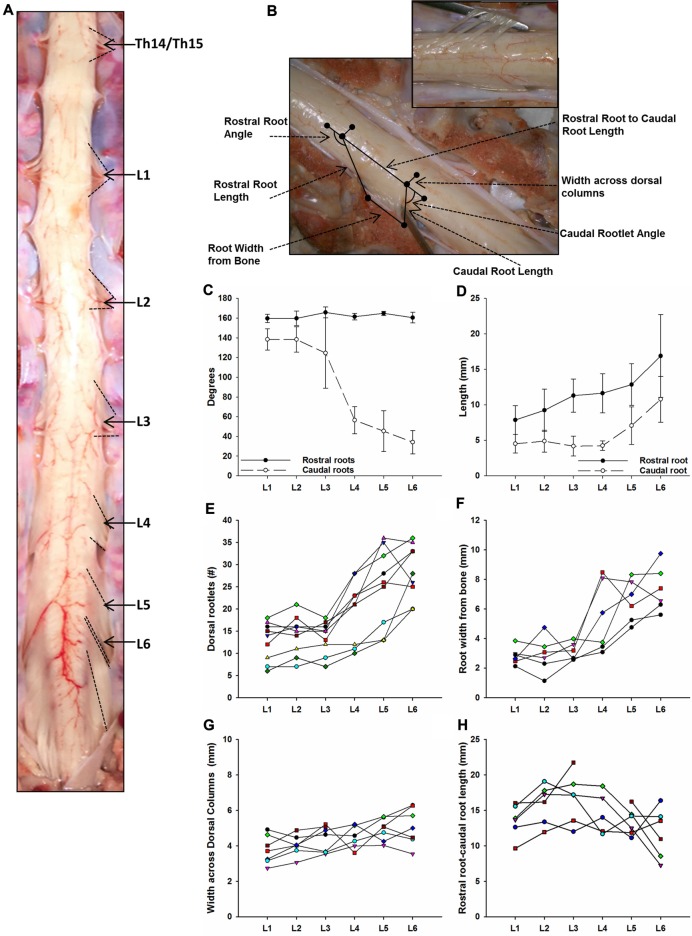
Dorsal root anatomy of the swine’s lumbar spinal cord. **(A)** Dorsal roots orientation in the swine lumbar spinal cord (Th14/15-L6 segments). Note the changes in orientation of angles, from rostral to caudal segments, denoted by dotted lines. Changes in dorsal root caudal angles are more evident in L4-L6. **(B)** Dorsal spinal cord anatomical measurements and dorsal rootlets count (inset). **(C)** Rostral (black dots) and caudal (white dots) dorsal root angles (mean ± SD; *n* = 5).** (D)** Rostral (black dots) and caudal (white dots) root lengths (Mean ± SD; *n* = 5). Data per specimen across lumbar segments is shown for: **(E)** number of dorsal rootlets (*n* = 9), **(F)** root width from bone (*n* = 6), **(G)** width across dorsal columns (*n* = 7) and** (H)** rostral root-caudal root length (*n* = 6).

The lumbosacral spine was extracted *en bloc* from each subject for dissection. Once extracted, landmarks were established along the facet joints, transverse processes, as well as anterior and posterior portions of the vertebral laminae as indicated on Figure 1A. The distances between these landmarks included: (a) intervertebral length; (b) midvertebrae foramen length (right and left); (c) intervertebral spinous process length; and (d) vertebral bone length (left and right). Anatomical bone landmarks were measured manually using slide calipers. A laminectomy was then performed across all lumbosacral vertebrae to expose the spinal cord. The dura was incised to expose the spinal cord, and anatomical landmarks were determined across lumbar segments as illustrated in Figure 1A and measured with slide calipers as follows: (a) transverse diameter at the dorsal root entry with reference at three locations: rostral, middle and caudal; (b) spinal cord segment length, from the caudal extent of a segment’s rootlet entry zone to the caudal extent of the next segment’s rootlet entry zone; (c) segment width at dorsal root entry zone; and (d) distance from midvertebrae foramen to dorsal rootlet entry. Next, high resolution pictures of each segment were taken with a surgical microscope (Leica M20, 4× objective) after which measurements were taken of the spatial orientation of the dorsal roots and rootlets (Figures 2A,B) using GeoGebra open source software^1^. Lumbar dorsal root and rootlets analysis included: (a) number of dorsal rootlets; (b) root width from bone; (c) rostral and caudal roots angles; (d) rostral and caudal rootlet length from bone; (e) width across dorsal columns; and (f) rostral root to caudal root length.

### EES Procedure

Two animals underwent *in vivo* electrophysiological experiments consisting of recording spinally evoked motor responses from select hind limb muscles during EES. The surgical approach has been described in detail previously (Hachmann et al., 2013). Intramuscular telazol (5 mg/kg) and xylazine (2 mg/kg) were administered for anesthesia induction and 1.5%–3% isoflurane for maintenance. Fentanyl was continuously administered during surgery (2–5 mg/kg/h) for analgesia. Briefly, laminectomies were performed to expose the lumbosacral spinal cord (L1-S1). Connective and fat tissue was removed keeping the dura mater intact. For EES, we used two types of electrodes: Subject 1 was tested with a single contact, custom-made spherical stainless steel electrode (2.5 mm diameter) and Subject 2 with an 8-contact stainless steel rod array (1.3 mm diameter, 3 mm contact length, 4 mm spacing between contacts; Model 3874, Medtronic, MN, USA). The spherical electrode was sequentially placed over the dorsal roots entry zones at L1, L2 and L3 segments as well as distally locations to the dorsal roots in intersegmental positions (L1-L2, L2-L3 and L3-L4. This approach allowed the electrode to be manipulated with ease in relationship to the dorsal spinal cord anatomy (i.e., dorsal rootlets). To study caudal segments we used the rod array that was placed on the midline spanning L4-L6 segments to cover most of the dorsal rootlets in that region, which are denser compared to the rostral segments, and therefore, an intersegmental distance between them cannot be identified (Figure 2A). A reference electrode was inserted in the paravertebral muscles on the right side of the surgical site. An isolated pulse generator (A-M systems, Sequim, WA, USA) delivered biphasic square wave pulses (500 μs pulse width) at 0.5 Hz with amplitudes ranging from 0.25 mA to 4.5 mA.

### Electrophysiological Recordings

To record spinally evoked motor responses, muscles of the hind limbs were dissected bilaterally and a pair of two stainless steel wires (AS 631, Cooner wire) were placed intramuscularly to capture electromyography (EMG) from the following muscles: gluteus maximus (GLU); rectus femoris (RF); vastus lateralis (VL); tibialis anterior (TA); soleus (SOL); and medial gastrocnemius (MG). Signals were amplified (Bio amplifier AD Instruments, Colorado Springs, CO, USA) and digitized (sampled at 4 KHz, hi-pass 0.5 Hz) using a PowerLab acquisition system (AD instruments, Colorado Springs, CO, USA). Offline, the recorded responses were band-pass filtered (20–500 Hz, Butterworth) in MatLab (The MathWorks Inc., Natick, MA, USA). To determine the onset and amplitude of each response, waveforms were analyzed and compared starting from the voltage threshold that elicited the onset of early (ER) and middle (MR) responses and continuing as stimulation intensity was incrementally increased in 0.25 mA steps (Lavrov et al., 2006). Peak-to-peak response amplitudes and latencies were measured in a window of 5–25 ms from stimulation artifact using custom MATLAB script. ER peak-to-peak amplitude was determined on the positive slope of the waveform and on the negative slope for MR. Examples of the peak-to-peak amplitude and latency measurements are depicted in the bottom traces of Figure 5A. If evoked responses were not distinguishable, latencies and amplitudes were measured at the first and second peaks in the same time window.

**Figure 3:**
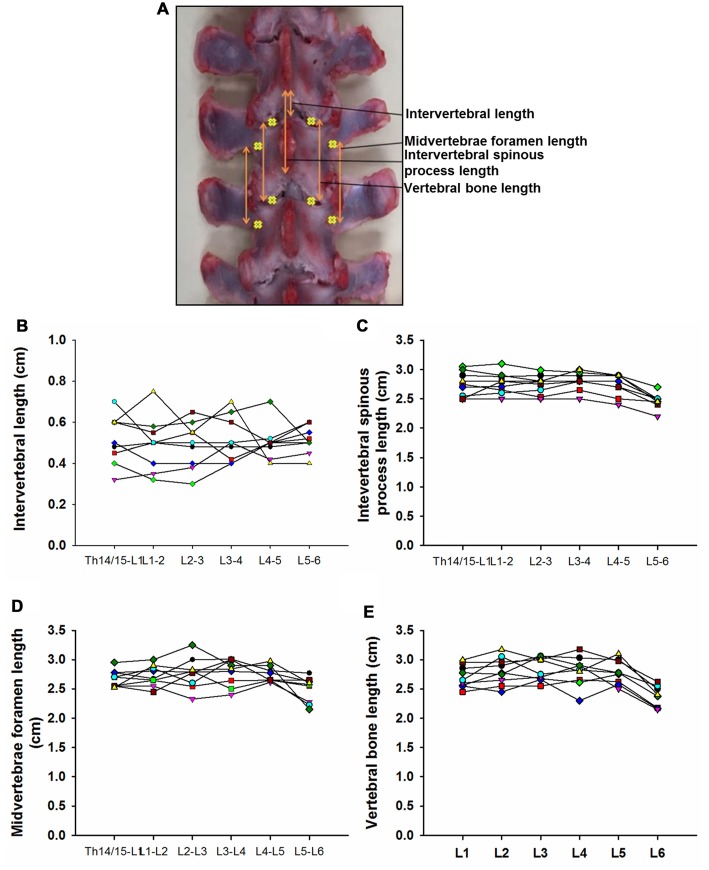
Swine’s lumbar spine anatomy. **(A)** Vertebral landmark measurements. Data per specimen (*n* = 9) across Th14/Th15-L1 to L5-L6 intersegments is shown for:** (B)** intervertebral length, **(C)** intervertebral spinous process lengths, **(D)** midvertebrae foramen length and **(E)** vertebral bone length.

**Figure 4:**
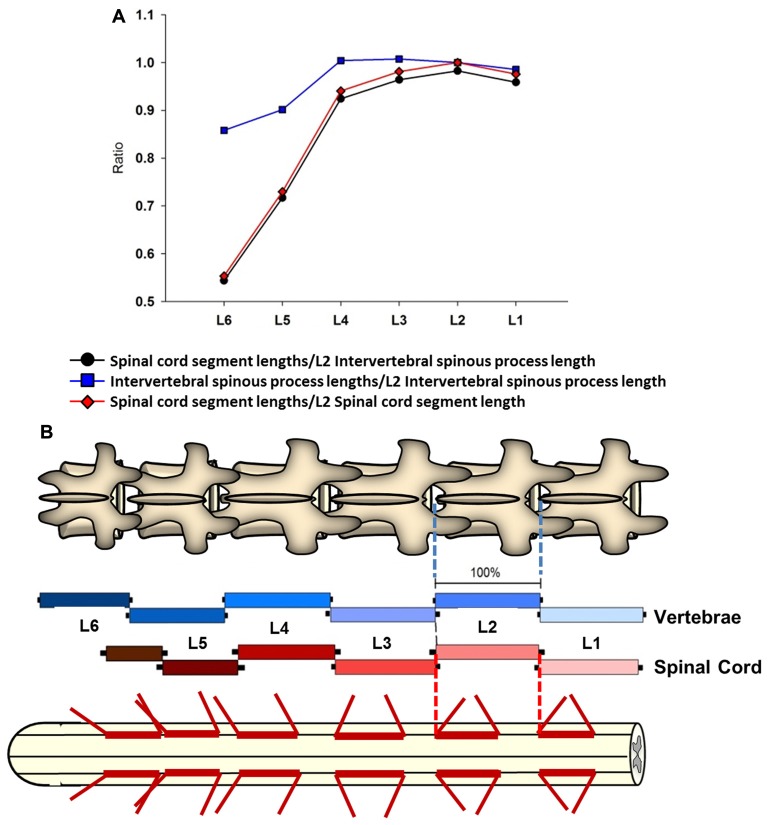
Intersegmental relationship between the spine and spinal cord. **(A)** Ratios between the intervertebral spinous process length at L2 vertebra and the spinal cord segments lengths from L1 to L6 vertebras (black line and circles). The ratios between the intervertebral spinous process lengths across lumbar segments and the L2 intervertebral spinous process length (blue line and squares), as well as the ratios between the spinal cord segments lengths and L2 spinal cord segment length are also shown (red line and diamonds). **(B)** Schematic representation of intervertebral spinous process lengths (top diagram and blue palette rectangles) and spinal cord segment lengths (bottom diagram and red palette rectangles) showing the segmental correspondence between them. Mean lengths (±SD, black bars) are expressed as percentage of the L2 intervertebral spinous process length (100%). The thick red lines on the spinal cord diagram represent the segment sizes at dorsal root entries expressed as percentage respect to L2 intervertebral spinous process length. Dorsal root mean angles (rostral and caudal) are also shown (thin red lines).

**Figure 5:**
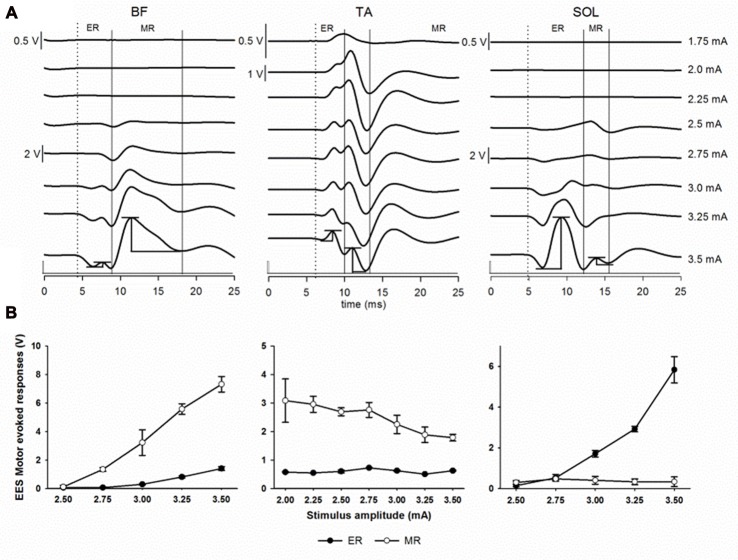
Epidural electrical stimulation (EES) evoked motor responses. **(A)** Early response (ER) and middle response (MR) representative responses recorded in BF, TA and SOL muscles at different stimulation intensities (1.75–3.5 mA) in subject 2 using the multi-contact rod array. Each trace is the average of ten motor evoked responses. Dotted lines indicate the beginning of the first deflection corresponding to ER. Continuous lines indicate MR. Examples of the peak-to-peak amplitude measurements for both ER and MR are shown at the bottommost traces (3.5 mA). **(B)** Recruitment curves showing ER (black dots) and MR (white dots) responses as shown in **(A)**. Note the different scales. Abbreviations: BF, Biceps femoris; TA, tibialis anterior and SOL, soleus.

### Data Analysis

SigmaPlot (Systat Software, San Jose, CA, USA) was used to perform statistical analysis. The Shapiro-Wilk method was used to determine if the data were normally distributed. If so, an Equal Variance Test was performed using the Brown-Forsythe method. Significant differences were determined by one-way repeated-measures analysis of variance (ANOVA). Pairwise multiple comparisons (Holm-Sidak) were performed to determine statistically significant differences between lumbar segments. Data that were not normally distributed were analyzed using Tukey test and pairwise multiple comparisons were done using Dunn’s method. Nine of the 11 swine used for this study were acquired post-mortem following unrelated experimental studies in which the spinal column and cord remained intact. The relationship between intersegmental measurements of the spine and spinal cord across lumbar segments was determined via linear regression. The following spine variables were correlated with the spinal cord segments lengths: intervertebral spinous processes, vertebral bone length and midvertebrae foramen lengths. Then, the highest correlation coefficient was used to determine a proper: (a) intersegmental vertebrae landmark; and (b) spine segment, and to use them as a reference to establish ratios between spine and spinal cord across segments. The means of the intersegmental landmark lengths (spine and spinal cord) were used to obtain the ratios. Once identified the proper intersegmental landmark and spinal segment (expressed as 100%, ±SD), a diagram illustrating the relationship between the spine and the spinal cord was performed. For this purpose, the length’s values of the intersegmental landmark and spinal cord were expressed as percentage (±SD) in relation to the spinal segment used as reference.

**Figure 6:**
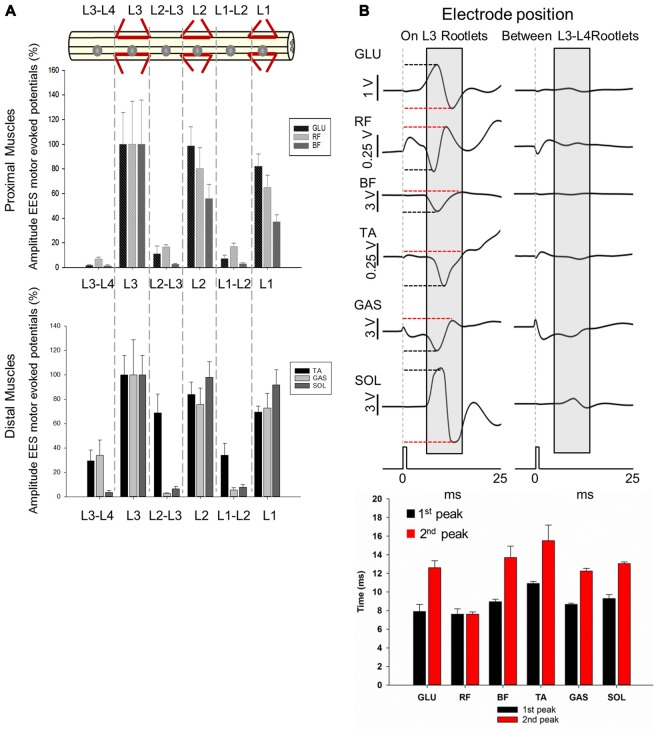
Motor responses during EES at L1-L3/L4. **(A)** Upper panel shows the electrode positions (single spherical electrode) over dorsal root entries zones (at L1, L2 and L3) and between segments (at L1-L2, L2-L3 and L3-L4) in subject 1. Amplitude of the motor evoked potentials is expressed as % (±SEM). Responses were recorded in proximal (upper plot) and distal muscles (bottom plot). **(B)** Representative averaged traces of motor potentials (gray rectangles, 10 ms time window) evoked at 1.4 mA. Each trace represents the average of ten motor responses. The electrode was placed on dorsal roots (L3, left traces) and between segments (L3-L4, right traces). Mean latencies (±SD) of the first (red bar) and second peak (black bar) are shown below the traces on left for each muscle. (See “Materials and Methods” Section, *Electrophysiological Recordings*, for details). Abbreviations: TA, tibialis anterior; GAS, medial gastrocnemius; SOL, soleus; GLU, gluteus; RF, rectus femoris and BF, biceps femoris.

**Figure 7:**
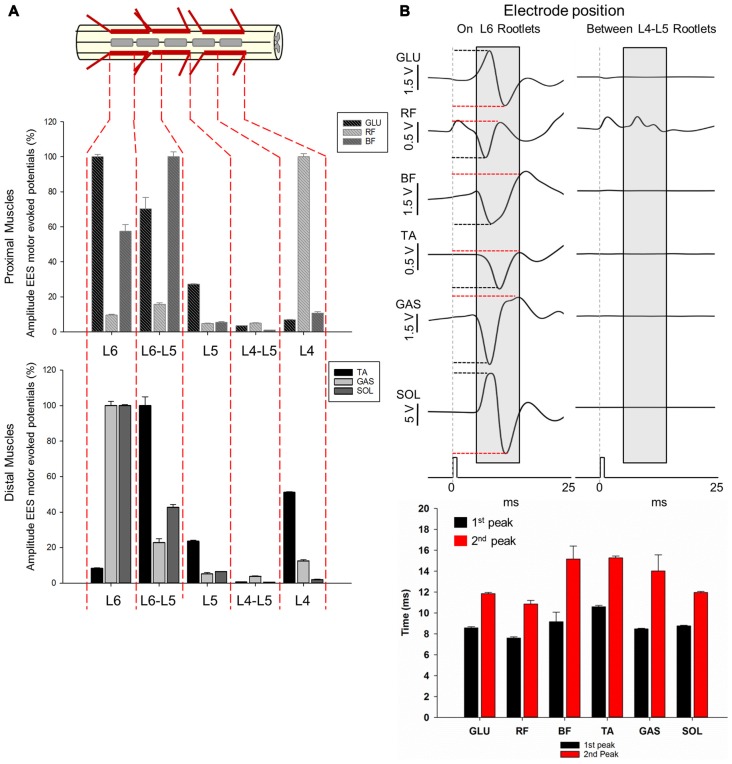
Motor responses during EES at L4-L6. **(A)** The multi-array rod electrode (8-contacts rod array, Model 3874, Medtronic, MN, USA) was placed on the midline of the spinal cord at the dorsal rootlets entry levels (L4, L5 and L6) and in approximate locations between the segments (L4-L5 and L5-L6) in subject 2. Gray rectangles in the upper diagram represent the relative position of the multi-array rod electrode. Amplitude of the motor evoked responses is expressed as % (±SEM). Responses were recorded in proximal (upper plot) and distal muscles (bottom plot). **(B)** Representative averaged traces of motor responses (gray rectangles, 10 ms time window) evoked at 1.4 mA. The electrode was located proximal to L6 dorsal root entry zone (left traces) and in the intersegmental location L4-L5 (right traces). Electromyography (EMG)’s of distal and proximal muscles were recorded. Each trace represents the average of ten motor responses. Mean latencies (±SD) of the first (red bar) and second peak (black bar) are shown below traces on left for each muscle. (See “Materials and Methods” Section, *Electrophysiological Recordings*, for details). Abbreviations: TA, tibialis anterior; GAS, medial gastrocnemius; SOL, soleus; GLU, gluteus; RF, rectus femoris and BF, biceps femoris.

Electrophysiological data (*n* = 2 male swine) were analyzed as follows: ten evoked responses were averaged for each stimulation trial and the highest amplitude values across lumbar segments were expressed as 100% for each muscle. Then, the rest of the amplitudes were expressed as % (±SEM) of the maximal value. Data sets were analyzed separately for each electrode used: spherical in L1-L3/L4 (Figure 6) and 8-contacts rod array in L4-L6 (Figure 7).

## Results

### Lumbar Spinal Cord Gross Anatomy

Lumbar spinal cord gross anatomical landmarks including transverse diameter, segment length, segment length at dorsal root entry and midvertebrae foramen to rootlets distance are illustrated in Figure 1A. Data (mean, ±SD) from these anatomical measurements are summarized in Table 1. Because no statistical differences were found between the three measured transverse diameters at dorsal root entry with respect to rostral, middle or caudal reference points (data not shown), we averaged them into a single diameter measurement per segment. Spinal cord transverse diameter was similar from L1 to L3, but increased at more caudal segments L4-L6, with longer diameter at L5 in 8/9 subjects (Figure 1B, Table 1). In fact, the transverse diameter from L4 to L6 was significantly higher than L1, as well as L5 compared to L2 (Table 2). The length of the spinal cord segments was similar from L1 to L3, and then gradually decreased from L4 to L6 (Figure 1C), with the L5 and L6 segments being significantly shorter than the L1-L4. Moreover, L6 was also significantly shorter than L5 (Table 2). In Figure 1D and Table 1, is shown that the segment width at the dorsal root entry was similar across all lumbar segments and no significant differences across segments were found (Table 2). The distance from the midvertebrae foramen to dorsal rootlets in L1-L4 was similar and then increased at L5 and L6 (Figure 1E, Table 1), being just L6 significantly higher than L1-L4 (Table 2). These results show significant anatomical differences at L4-L6 segments compared with a relatively similar anatomy in L1-L3 segments. These differences are primarily characterized by an increase in spinal cord diameter and a decrease in segment length in L4-L6, as well as an increase in the distance from the midvertebrae foramen to the dorsal rootlets at the same segments, with a significantly higher distance at L6.

**Table 1:** Spinal cord measurements.

	L1	L2	L3	L4	L5	L6
SCD	0.75 ± 0.10	0.78 ± 0.080	0.81 ± 0.08	0.90 ± 0.08	0.94 ± 0.12	0.89 ± 0.09
SLcc	2.62 ± 0.20	2.69 ± 0.28	2.64 ± 0.28	2.53 ± 0.30	1.93 ± 0.33	1.48 ± 0.29
SLDRE	0.47 ± 0.06	0.50 ± 0.09	0.51 ± 0.11	0.54 ± 0.09	0.56 ± 0.10	0.53 ± 0.07
MFR	1.25 ± 0.19	1.19 ± 0.24	1.21 ± 0.26	1.22 ± 0.14	1.58 ± 0.29	2.17 ± 0.29

*Numbers are in cm (±SD). Nomenclature: SCD, Spinal cord transverse diameter; SLcc, Spinal cord segment length caudal-to-caudal; SLDRE, Segment length at dorsal root entry; MFR, Midvertebrae foramen to rootlets length.* n* = 9*.

**Table 2:** Anatomical spinal cord comparisons across spinal segments.

Anatomical landmark		
SCD	L4	L1, *p* < 0.05
	L5	L1, L2, *p* < 0.01
	L6	L1, *p* < 0.05
SLcc	L5	L1, L2, L3, L4, *p* < 0.001; L6, *p* < 0.05
	L6	L1, L2, L3, L4, *p* < 0.001
SLDRE		ND
MFR*	L6	L1, L4, *p* < 0.01; L2, L3, *p* < 0.001

ANOVA test. All pairwise multiple comparison (Holm-Sidak method). *Tukey test, all pairwise multiple comparison. Nomenclature: SCD, Spinal cord transverse diameter; SLcc, Spinal cord segment length caudal-to-caudal; SLDRE, Segment length at dorsal root entry; MFR, Midvertebrae foramen to rootlets length. ND, No statistical differences were found.

### Lumbar Dorsal Root and Rootlets (Root Fibers) Anatomy

As shown in Figure 2A, the dorsal roots vary in orientation across lumbar segments. Anatomical measurements of the dorsal roots and rootlets are illustrated in Figure 2B and included: number of dorsal rootlets, root width from bone, rostral and caudal root angles, rostral and caudal root lengths, width across dorsal columns and rostral root-caudal root length. Data (mean, ±SD) from dorsal roots and rootlets anatomy are listed in Table 3. In order to facilitate the comparison between rostral and caudal dorsal root angles, as well as rostral and caudal root lengths from bone, and to emphasize the anatomical differences across lumbar segments (for example, Figure 2A), means (±SD) are plotted in corresponding Figures 2C,D for seven specimens. While the rostral root angles from L1 to L6 did not vary significantly from one another, the caudal angles showed significant differences (Figure 2C, Table 3), being L5 smaller than those at L1 and L2 an L6 smaller than L1-L3 (Table 4). Both rostral and caudal root lengths were found similar in L1-L4 (Figure 2D, Table 3) and then they increased significantly at L5-L6 (Table 4). In Figures 2E–H, data per specimen are presented to show variability across animals. The number of dorsal rootlets was consistent across the L1, L2 and L3 segments and gradually increased from L4 to L6 (Figure 2E, Table 3), being the number of dorsal rootlets higher at L5 compared to L1 and L6 compared to L1-L3 (Table 4). We also found a gradual increase in dorsal root width from bone across spinal cord segments (Figure 2F, Table 3). In fact, the dorsal root width from bone at L5 was higher compared to L1 as well as L6 compared to L1 and L2 (Table 4). The width across the dorsal columns was generally uniform from L1 to L4, and then exhibited an increase at L5 and L6 (Figure 2G); however, no statistical differences across spinal segment were found (Table 4). No significant differences in rostral-root to caudal-root distance across lumbar segments were found even though there was a trend towards higher values at L2-L4 when compared to L1 and L5-L6 (Figure 2H, Table 4). Altogether, neuroanatomical measurements showed non-homogenous morphometric characteristics when comparing L1-L3 and L4-L6. The changes in the most caudal spinal cord segments L4, L5, and L6, included higher number of dorsal rootlets and sharper dorsal roots angles as well as an increase in the rostral and caudal root lengths and root with from bone.

**Table 3:** Dorsal spinal cord measurements.

	L1	L2	L3	L4	L5	L6
rRA (°), *n* =7	159.72 ± 4.19	159.8 ± 7.43	165.81 ± 5.47	161.48 ± 3.42	164.95 ± 2.05	160.55 ± 5.45
cRA (°), *n* =7	138.43 ± 10.79	138.33 ± 12.88	124.62 ± 35.7	56.78 ± 13.78	45.43 ± 20.77	34.13 ± 11.72
rR (mm), *n* =7	7.84 ± 2.03	9.24 ± 2.99	11.31 ± 2.33	11.64 ± 2.77	12.86 ± 5.84	16.89 ± 5.84
cR (mm), *n* =7	4.52 ± 1.31	4.89 ± 1.54	4.18 ± 1.40	4.26 ± 0.68	7.08 ± 2.64	10.79 ± 3.24
DR (#), *n* =9	15.11 ± 6.49	15.78 ± 4.23	15.0 ± 4.18	23.55 ± 10.45	26.22 ± 8.39	30.22 ± 5.69
rWB (mm), *n* =6	2.85 ± 0.80	2.96 ± 0.57	3.27 ± 0.73	5.43 ± 0.64	6.13 ± 0.62	6.87 ± 1.04
DC (mm), *n* =7	3.77 ± 0.59	4.03 ± 0.39	4.38 ± 0.55	4.46 ± 0.40	4.92 ± 0.52	5.09 ± 0.61
rR-cR (mm), *n* =6	12.94 ± 0.32	14.43 ± 0.47	16.12 ± 0.26	14.19 ± 0.14	12.97 ± 0.27	12.76 ± 0.59

*Nomenclature: rRA, rostral root angle; cRA, caudal root angle; rR, rostral root length; cR, caudal root length; DR, dorsal rootlets; rWB, root width from bone; DC, width across dorsal columns; rR-cR, rostral root to caudal root length*.

**Table 4:** Anatomical dorsal spinal cord comparisons across spinal segments.

Anatomical landmark		
rRA		ND
cRA*	L5	L1, L2, *p* < 0.05
	L6	L1, L2, L3, *p* < 0.01
rR*	L6	L1, *p* < 0.01; L2, *p* < 0.05
cR	L5	L1, L3, L4, *p* < 0.05; L6, *p* < 0.01
	L6	L1, L2, L3, L4, *p* < 0.001;
DR	L5	L1, *p* < 0.05
	L6	L1, L2, L3, *p* < 0.01
rWB*	L5	L1, *p* < 0.05
	L6	L1, *p* < 0.01 L2, *p* < 0.05
DC		ND
rR-cR		ND

ANOVA test. All pairwise multiple comparison (Tukey test). *All pairwise multiple comparison (Dunn test). Nomenclature: DR, dorsal rootlets; rWB, root width from bone; rRA, rostral root angle; cRA, caudal root angle; rR, rostral root length; cR, caudal root length; DC, width across dorsal columns; rR-cR, rostral root to caudal root length. ND, No statistical differences were found.

### Spine Anatomical Landmarks

Data from intersegmental spine landmarks are summarized in Table 5 and plotted in Figure 3, these measurements include the following lengths: intervertebral, intervertebral spinous process, midvertebrae foramen and vertebral bone (Figure 3A). Intervertebral length across lumbar segments is plotted in Figure 3B per specimen (*n* = 9). This anatomical landmark was not statistically different across lumbar segments (Table 6). Intervertebral spinous process length at L5-L6 was found to be shorter compared to L2-L3, L3-L4 and L4-L5 in all specimens (*n* = 9) as shown in Figure 3C and Table 6. In Figure 3D, midvertebrae foramen length is shown per specimen (*n* = 9). This anatomical landmark did not vary significantly across lumbar segments except for L5-L6 which were found to be the shortest (Table 6). Vertebral bone length was similar from L1 to L4, although it was slightly higher at L4 in 8/9 specimens (Figure 3E), but then decreased significantly at L6 (Table 6). Except for the intervertebral length, the rest of the intersegmental spine landmarks exhibited a decrease in length at the most caudal segments (L5-L6).

**Table 5:** Intersegmental spine measurements.

	Th14/Th15-L1	L1-L2	L2-L3	L3-L4	L4-L5	L5-L6	L6-S1
IL	0.51 ± 0.11	0.49 ± 0.13	0.49 ± 0.11	0.51 ± 0.11	0.50 ± 0.08	0.48 ± 0.13	0.47 ± 0.08
ISPL	2.69 ± 0.15	2.73 ± 0.11	2.75 ± 0.20	2.75 ± 0.16	2.46 ± 0.15	2.35 ± 0.14	
MVFL	2.67 ± 0.16	2.72 ± 0.19	2.76 ± 0.26	2.78 ± 0.23	2.74 ± 0.15	2.49 ± 0.22	
		L1	L2	L3	L4	L5	L6
VBL		2.66 ± 0.16	2.76 ± 0.19	2.79 ± 0.19	2.77 ± 0.25	2.74 ± 0.18	2.38 ± 0.36

*Numbers are in cm (± SD). Nomenclature: IL, intervertebral length; ISPL, Intervertebral spinous process length; MVFL, Midvertebrae foramen length; VBL, vertebral bone length.* n* = 9*.

**Table 6:** Anatomical intersegmental spine comparisons across spinal segments.

Anatomical landmark		
IL		ND
MVFL	L4-L5	L5-L6, *p* < 0.01
	L5-L6	L1-L2, *p* < 0.05; L2-L3, *p* < 0.01; L3-L4, *p* < 0.001
ISPL	L4-L5	L5-L6, *p* < 0.05
	L5-L6	L2-L3, L3-L4, *p* < 0.05
VBL	L6	L1, *p* < 0.01, L2, L3, L4, L5, *p* < 0.001

*ANOVA test. All pairwise multiple comparison (Holm-Sidak method). Nomenclature: IL, intervertebral length; MVFL, Midvertebrae foramen length; ISPL, Intervertebral spinous process length; VBL, vertebral bone length. ND, No statistical differences were found*.

### Relationship between Anatomical Landmarks of the Spine and Spinal Cord

A linear regression analysis was performed to examine the intersegmental relationships between the spinal cord and the spine using the following anatomical landmarks: spinal cord segments lengths (Figure 1C, Table 1), midvertebrae foramen, intervertebral spinous process, and vertebrae bone length (Figures 3C–E, Table 3). Correlation coefficients are listed in Table 7. The strongest correlations were found between the length of the intervertebral spinous process and the length of the lumbar spinal cord segments, particularly at L2 (*r* = 0.904) and L4 (*r* = 0.858). However, the correlation between the vertebral bone length and the spinal cord segment length was weak as well as the correlation between the midvertebrae foramen and the spinal cord segment length (see Table 7). Due to the high correlation between the intervertebral spinous process length and the spinal cord length at L2, we used this segment as a reference to establish an intersegmental anatomical relationship between the spine and the spinal cord. Then, ratios were established between the mean of the spinal cord segment length across lumbar segments and the mean of L2 intervertebral spinous process length (Figure 4A, black line and circles). Ratios were similar around the rostral segments (L1, 0.95; L2, 0.98; L3, 0.96 and L4, 0.92) but were lower at L5 and L6 (0.71 and 0.54, respectively). As a reference, the ratios between the mean of the intervertebral spinous process length across segments and the mean of L2 intervertebral spinous process length (blue line and squares: L1, 0.98; L2, 1.00; L3, 1.00; L4, 1.00, L5, 0.90 and L6, 0.86) as well as the ratios between the mean of the spinal cord segment length across segments and the mean of L2 spinal cord segment length (red line and diamonds: L1, 0.96; L2, 1.00; L3, 0.96; L4, 0.94; L5, 0.73 and L6, 0.55) were included (Figure 4A). In Figure 4B, diagrams represent the spine (top) and spinal cord (bottom) intersegmental relationship. The blue and red palettes rectangles indicate the intervertebral spinous process lengths and spinal cord segmental lengths, respectively. The L2 segment was selected as a reference as indicated above. Then, the L2 mean intervertebral spinous process length was defined as 100% and intervertebral spinous processes lengths and spinal cord segmental lengths were defined as a percentage with respect to L2. Note that the length of the spinal cord in relation to the vertebrae in the rostral segments (L1 to L3) tends to be similar, while the spinal cord shortening is evident in the L4-L6 segments. Among the spine landmarks described in this study, the intervertebral spinous process length, specifically at segment L2, could be used to establish an anatomical segmental relationship between the spine and the spinal cord in order to target dorsal spinal structures based on vertebral bones landmarks.

**Table 7:** Linear correlation analysis between vertebrae bone and spinal cord.

Spinal level	ISP-SLcc	VB-SLcc	MVF-SLcc
L1	0.711	0.359	0.263
L2	0.904	0.329	0.161
L3	0.541	0.482	0.100
L4	0.858	0.087	0.217
L5	0.204	0.335	0.469
L6	0.236	0.366	0.286

*Numbers are coefficients (r). Nomenclature: ISP, Intervertebral spinous process length; VB, vertebral bone length; MVF, Midvertebrae foramen length; SC, spinal cord segment length (caudal to caudal) * n* = 9*.

### Functional Neuroanatomy of the Lumbosacral Spinal Cord

Next, we evaluated the relationship between the EES motor evoked potentials and the neuroanatomy of the spinal cord. We evaluated the amplitude and latency of spinally evoked motor responses during EES in proximal sites to the dorsal root entry zones and in distal sites at intersegmental locations across the lumbar segments. In Figure 5A, representative examples of spinally evoked ER and MR in proximal (BF) and distal (TA and SOL) muscles in Subject 2 are presented. EES was delivered through electrode contacts located close to the dorsal root entry zone at L5 (BF and SOL) and L6 (TA). Visible motor evoked responses appeared at 2.5 mA in BF and SOL and at 1.75 mA in TA. Averaged latencies were determined for ER (BF, 7.97 ± 0.57 ms; TA, 8.80 ± 0.30 ms and SOL, 9.94 ± 0.69 ms) and MR (BF, 11.77 ± 0.71 ms; TA, 10.74 ± 0.27 ms and SOL, 14.10 ± 1.25 ms). Recruitment curves showing the stimulus amplitude (mA) and the mean (in volts) of the EES motor evoked potentials (±SD) of both ER and MR for the same muscles are depicted in Figure 5B. While in these examples the ER and MR are clearly defined, the difference between ER and MR in other muscles at different contact locations was not clearly distinguishable (i.e., on Figures 6, 7). At the same time, the amplitudes and latencies of the EES motor evoked potentials were corresponding to the first and second peaks (similar to ER and MR) and were measured in a time window similar to the examples shown in Figure 5A.

### Spinally Evoked Motor Responses from Proximal and Distal Lumbar Segments

#### Proximal Segments (L1-L4)

Higher amplitudes were consistently observed for both proximal and distal muscles when EES (2.5 mA stimulus amplitude) was delivered over the dorsal root entry zone within segments (L1, L2 and L3) compared to when stimulated in between the segments (L1-L2, L2-L3 and L3-L4; Figure 6A). Maximal amplitudes of responses were expressed as 100% (±SEM) and were found when stimulating at L3 for proximal (GLU, ±25.96%; RF, ±34.86% and BF, ±35.75%) and distal muscles (TA, ±15.92%; GAS, ±28.83% and SOL, ±15.89%). On the other hand, lowest amplitude responses occurred in proximal (GLU, 1.78 ± 0.39%; RF, 7.08 ± 1.42%; BF, 1.43 ± 0.64%) and distal (TA, 29.35 ± 8.86%; SOL, 3.44 ± 1.67%) muscles when EES was delivered at L3-L4, with the exception of GAS for which the lowest response was recorded when stimulating at L2-L3 (3.44 ± 1.67%).

EES responses in GLU evoked stimulating over the dorsal root entry zone at L1, L2 and L3 were higher compared to stimulation delivered distally from dorsal root entry zones at L1-L2, L2-L3 and L3-L4 (*p* < 0.05). RF motor responses in L1 and L3 were higher just compared to L3-L4 (*p* < 0.001) while motor responses evoked in L2 were higher compared to all intersegmental electrode positions: L1-L2, L2-L3 (*p* < 0.05) and L3-L4 (*p* < 0.001). Similarly to GLU, BF exhibited higher amplitudes when stimulating at L1 compared to L2-L3 (*p* < 0.05) and L3-L4 (*p* < 0.001) as well as stimulation in L2 compared to L1-L2, L2-L3 (*p* < 0.05) and L3-L4 (*p* < 0.001) and L3 compared to L1-L2, L2-L3 (*p* < 0.05) and L3-L4 (*p* < 0.001). A similar pattern was observed in distal muscles. Motor responses in TA evoked by stimulation over the dorsal root entry zone at L2 were higher compared to L1-L2 and L3-L4 (*p* < 0.05) as well as L3 compared to L1-L2 (*p* < 0.05) and L3-L4 (*p* < 0.001). Motor responses however, were not higher compared to L2-L3, where TA had a relatively high amplitude response (68.78 ± 15.56%) as shown on Figure 6A. Amplitudes obtained in GAS when stimulating over dorsal root entry zone at L1, L2 and L3 were higher compared to L1-L2 (*p* < 0.05) and L2-L3 (*p* < 0.001) but not to L3-L4. SOL motor potentials evoked were also higher compared to L3-L4 (*p* < 0.05). Finally, SOL exhibited higher amplitudes in L1, L2 and L3 compared to all intersegmental electrode positions (L1-L2, L2-L3 and L3-L4, *p* < 0.05, being *p* < 0.001 in L2 compared to L3-L4).

These results show that EES-induced motor responses are highly dependent upon position of the electrode over the dorsal roots entry zone. Shifting the electrode just a few millimeters away from that area, for example from L1 to L1-L2, leads to a significant increase in motor thresholds, while shifting the electrode a few centimeters, for example from L1 to L3 segment, causes no significant difference in evoked responses in both proximal and distal muscles. Particularly, maximal peak-to-peak amplitudes were observed when EES was delivered at L3 in all recorded muscles and no significant differences were found when comparing amplitudes with those in L1 or L2 (Figure 6A), where electrode was located ≈5.2 cm and ≈2.6 cm apart, respectively. A dramatic decrease in amplitudes was observed when EES was applied at intersegmental locations compared with stimulation over the dorsal root entry zone in nearby electrode locations. For instance, the difference in amplitudes when EES was delivered at L3 and L3-L4 was up to 90% in proximal and 60% in distal muscles (Figure 6A), considering that the distance between L3 and L3-L4 electrode positions was approximately 1 cm. In Figure 6B, examples of 10 averaged EES-evoked motor responses applying 1.4 mA are shown for proximal and distal muscles. Note the difference in amplitudes between evoked responses when the electrode was placed over the dorsal root entry at L3 (highest amplitudes, 100%) and between L3-L4 segments, distally from dorsal rootlets (lower amplitudes). Mean latencies (±SD) of the first and second identified peaks (on L3 rootlets) are plotted in the bottom panel on Figure 6B.

#### Distal Segments (L4-L6)

Amplitudes and latencies of EES evoked motor potentials in L4-L6 were investigated using the multi-contact rod array placed on the midline of the spinal cord in subject 2 (Figure 7). As shown in Figures 1, 2, L4-L6 segments exhibit significant anatomical differences compared with the relatively invariant rostral segments L1-L3. The shortening of caudal segments is accompanied with an increasing number of dorsal roots and a change in angles as they enter the spinal cord (Figures 1C, 2C,E). These anatomical differences preclude determining intersegmental positions clearly, as distance between dorsal roots is smaller, especially from L5 to L6 (≈0.19 cm). Positioning of electrode contacts was related to the proximity to dorsal root entry zone (L4, L5 and L6) and neighboring intersegmental locations (L4-L5 and L5-L6). Highest amplitudes during EES-evoked responses were expressed as 100% (±SEM). In general, maximal amplitudes were evoked delivering EES at the most caudal electrode locations: L5-L6 in BF (±2.7%) and TA (±4.99%) and L6 in GLU (±1.4%), GAS (±2.46%) and SOL (±0.52%). The exception was RF, which exhibited maximal amplitude (100%) when EES was delivered at L4 (±1.17% as shown in Figure 7. On the other hand, EES at L4-L5 evoked the lowest amplitudes in GLU (3.50 ± 0.05%), BF (0.97 ± 0.04%), TA (0.62 ± 0.05%), GAS (3.85 ± 0.32%) and SOL (0.48 ± 0.04%). RF exhibited the lowest amplitude at L5 (4.86 ± 0.04%) but not significantly different from that in L4-L5 (4.92 ± 0.35%) as shown in Figure 7. As shown in the previous section, lowest amplitudes at L4-L5 can be attributed to the fact that the contact position was located in an intersegmental location, distally from dorsal rootles. The distance between these segments was about 0.67 cm. (see for example Figure 2A and diagram on Figure 4B for comparison with intersegmental distance between L5 and L6). Multiple comparisons showed that the amplitude of the GLU motor response when stimulating at the most caudal electrode positions L5, L5-L6 and L6 were higher compared to more rostral segments. In fact, higher amplitudes were evoked in L6 compared to L4-L5, L4 (*p* < 0.001) and L5 (*p* < 0.05). Stimulation in the adjacent position L5-L6, produced higher amplitudes compared to that in L4-L5 (*p* < 0.001) and L4 (*p* < 0.05). Albeit stimulation at L5 evoked a small amplitude (27.26 ± 0.12%) compared to that in more caudal segments L5-L6 (70.24 ± 6.59%) and L6 (100 ± 1.46%), EES in L5 evoked higher amplitudes than that in L4-L5 (*p* < 0.05). Evoked responses in BF stimulating at L4 produced higher amplitudes compared with adjacent position L4-L5 (*p* < 0.05). EES at L5-L6 exhibited higher amplitudes than the intersegmental position at L4-L5 (*p* < 0.001) but also compared to L5 (*p* < 0.01) and L4 (*p* < 0.05). Also, EES at L6 produced higher amplitudes compared to L4-L5 (*p* < 0.001) and L5 (*p* < 0.05). The amplitude of the RF motor potentials when delivering EES at L4 was higher compared to that in L4-L5, L5 (*p* < 0.001) and L6 (*p* < 0.05). Moreover, amplitude of the motor response evoked at L5-L6 was also higher than that in L4-L5 and L5 (*p* < 0.05). Similar results were obtained for distal muscles, where EES at caudal electrode positions (L5-L6 and L6) evoked higher amplitudes than motor potentials stimulating at more rostral electrode positions (L4, L4-L5 and L5). The exception was the amplitude in TA when EES was delivered at L4, being 51.09 ± 0.42% with respect of the maximal amplitude (100%) evoked at L5-L6 (Figure 7A). The amplitude of TA motor potentials evoked at L5-L6 was higher compared to L4-L5, L6 (*p* < 0.001) and L5 (*p* < 0.05) as well as EES at L4 and L5 compared to amplitudes recorded in the adjacent contact location L4-L5 (*p* < 0.001 and *p* < 0.05 respectively). EES at L4 was also higher compared to L6 (*p* < 0.05). Similar motor response amplitudes were recorded in GAS and SOL with EES at L6 (100% in both muscles). EES at L6 in GAS evoked higher amplitudes than those in L4-L5, L5 (*p* < 0.001) and L4 (*p* < 0.05), similar pattern was observed in SOL (L6 vs. L4-L5, L4, *p* < 0.001 and vs. L5 *p* < 0.05). Moreover, stimulation in the adjacent electrode position L5-L6 in GAS and SOL evoked higher amplitudes compared to L4-L5 (*p* < 0.001). EES in the same intermediate electrode position L5-L6 produced higher amplitudes in GAS compared to L5 (*p* < 0.05) and in SOL compared to L4 (*p* < 0.05). Finally, motor responses in SOL evoked by stimulation at L5, produced higher amplitudes compared to those at L4-L5, (*p* < 0.05). Representative examples of ten averaged EES motor responses evoked at 1.6 mA are shown for proximal and distal muscles in Figure 7B. Mean latencies (±SD) of the first and second identified peaks (EES was delivered at L6) are shown in the bottom panel on Figure 7B. Overall, results of functional neuroanatomy of lumbar segments show that the peak-to-peak amplitude of EES evoked motor responses across lumbar segments is highly dependent upon the proximity of the electrode to the dorsal rootlets entry zone and less dependent to the position of the electrode across different spinal segments, regardless of whether they were evoked by the spherical single electrode (Figure 6) or the multi-contact rod array (Figure 7).

## Discussion

EES has showed positive and encouraging results in SCI patients (Harkema et al., 2011; Angeli et al., 2014; Grahn et al., 2017). Computational studies (Holsheimer and Struijk, 1991; Struijk et al., 1993; Holsheimer, 1998) and experimental results in rodents (Capogrosso et al., 2013) have shown that the spinal cord neuroanatomy is an important factor that influences motor responses enabled via EES; however, a suitable intermediary animal model for EES aimed to improve the current strategies following SCI deserves particular attention (Hachmann et al., 2013; Kowalski et al., 2013; Schomberg et al., 2017). Here, for the first time we provide an evidence of the influence of electrode position in relation to the dorsal roots spatial orientation on effect of EES.

### Anatomy of the Swine Lumbar Spinal Cord

Relevant anatomical landmarks in bony structures and spinal cord are described in this study (Figures 1–3). Still, anatomical differences between human and swine spinal cord should be taken into account, for example, the number of lumbar vertebrae (5 in human and 6 in swine) and some vertebral morphometric characteristics (Dath et al., 2007). To our knowledge, there are no detailed anatomical descriptions of the spatial orientation of dorsal elements of the spinal cord in any large animal model. In terms of the spinal cord anatomy, the length of the spinal cord in humans terminates at L1-L2 while the swine spinal cord continues to lower lumbosacral segments (Watson et al., 2008). In addition, segment length in the swine (Figure 1C; Table 1) is also longer compared to humans (Ko et al., 2004). The number of dorsal rootlets across the spinal cord in human cadavers was measured by Kirazli et al. (2014). These authors found that at lumbar segments, the number of dorsal roots ranged between 4.7 ± 0.6 at L5–6.7 ± 1.2 at L3. In the swine, the highest values were found, ranging from 15.0 ± 4.18 at L3, to 30.22 ± 5.69 at L6 (Figure 2D). In the swine, we also found significant anatomical differences in L5 and L6 segments compared to L1-L4 segments (Figures 1, 2; Tables 1, 2). The shortening of the spinal cord in L5-L6 is related with an increase in the midvertebrae foramen to rootlets distance (Figure 1D). A decrease in L5-L6 caudal root angles (Figure 2C) and an increase in the root-to-root length in these segments were also found (Figure 2E). A significant increase in rostral and caudal root length and root width from bone in L6 compared to L1-L3 was also observed (Figure 2F). Spine morphometric differences are not only observed in the swine, but also in different large animals used as translational models to assess vertebral biomechanical properties, spine pathology and surgical implantation techniques; therefore, specific anatomical differences should be noticed according to the purpose of the research (McLain et al., 2002; Busscher et al., 2010; Sheng et al., 2010, 2016; Engelke et al., 2016). Another important aspect of the swine model that has to be considered is the weight (size) of the animals as conventional breeds can reach more than 100 kg typically at 4 months. In this study we used swine with similar weight (25–40 kg) as reported in articles describing vertebral morphometry (Bozkus et al., 2005; Busscher et al., 2010), SCI model (Lee et al., 2013), feasibility of EES as a restorative paradigms (Hachmann et al., 2013; Guiho et al., 2017) and stereotactic-guided micro stimulation (Grahn et al., 2016). For chronic experiments with EES, minipigs breeds might be considered as an option in terms of their growth and handling. Yet, additional data on EES in effect of the neuroanatomy in acute experiments performed on swine or minipigs should be collected in order to expedite technologies directed toward effective therapeutic strategies. The study of the effect of EES in relation to the dorsal neuroanatomy provided here contributes to filling the gaps regarding the swine as a large animal model during neuromodulation strategies.

### Intersegmental Correlation between Vertebrae and Spinal Cord

In this study, we measured intersegmental vertebral landmarks (Figure 3) instead of measuring anatomical dimensions in isolated vertebrae (McLain et al., 2002). After performing linear regression analysis (Table 4), high correlation coefficients between the intervertebral spinous process lengths and the spinal cord segment lengths were found, particularly at L2 (*r* = 0.9). This approach denoted similarities between L1-L3 segments and the shortening of the spinal cord in relationship to the spine at segments L1-L4. For this reason, L2 intervertebral spinous process length was selected as segmental reference to establish ratios between the spine and spinal cord and to develop a model of the spinal cord including features of the dorsal root anatomy (Figures 4A,B). Correlations between intersegmental spine and spinal cord landmarks could be used in preclinical surgical maneuvers where the swine is used as a preferred animal model (Pleticha et al., 2013; Kowalski et al., 2016). Moreover, the anatomical data and particularly intersegmental correlations provided here could be combined with imaging studies in order to develop a 3D-model of the spinal cord to help in the implementation of specific targeting during implantation procedures (Kettler et al., 2007; Grahn et al., 2016).

### EES Evoked Motor Responses in the Swine Model

Initially described on rodent, three types of responses are observed during EES: ER, related with direct activation of motor fibers (latency about 3–5 ms); MR with latency (5–9 ms) associated with the activation of muscles afferents (group I and group II) and with formation of EMG bursts, and late response (LR) with a latency more than 10 ms (Lavrov et al., 2006) related with functional recovery of spinal cord circuitry after SCI (Lavrov et al., 2008). Murg et al. (2000) reported MR latencies from 9 ms to 17 ms in SCI patients during EES, while Sayenko et al. (2014) reported latencies in a range between 6 ms to 20 ms. Higher latencies ranging between 15.6 ± 2.9 ms (RF) to 31.0 ± 3.6 ms (flexor digitorum brevis) described as monosynaptic responses were evoked in healthy individuals during percutaneous electrical stimulation (Courtine et al., 2007). Discrepancy in latencies can be attributed to different placement of electrodes in relation of spinal structures (EES vs. percutaneous electrical stimulation), segmental electrode location, and distinction between ER and MR in evoked motor potentials. Here, for the first time we described the characteristics of the EES evoked motor responses in terms of amplitude and latency in the swine model (Figures 5–7). In additional EES experiments, latencies and histological sections of the motor columns could be combined in order to determine the location of the motor pools as shown in different species, but surprisingly not in the swine.

The latencies of EES evoked motor responses were found to be close to the human. Figure 5A clearly shows ER and MR, however, in many cases it was not easy to distinguish between both components, similar to what was also described in human studies (Sayenko et al., 2014). Additional analysis performed based on latency (window 5–10 ms after stimulus), showed two mean latencies that likely represent ER and MR in proximal and distal muscles (Figures 6B, 7B). Amplitude modulation of ER in this study can be explained based on the following: (a) the current generated by EES flows through the less resistive medium (cerebrospinal fluid), recruiting a higher number of motor axons as the stimulation intensity is increasing (Rattay et al., 2000); (b) the proximity of the stimulating electrode to dorsal roots, hence to ventral roots, generates an electrical field that could reach the motor axons relatively ease, however, anatomical data of the ventral roots will be required for precise comparison; and (c) antidromic activation through thick afferent fibers (i.e., group Ia) could also contribute in some extent to ER amplitude, although experiments and computational simulations in rats blocking synaptic transmission using TTX, showed direct activation of motor axons during EES suggesting that this explanation is unlikely (Capogrosso et al., 2013). Using increasing stimulation frequencies and vibration of the Achilles tendon could be used in future experiments to evaluate the changes of MR amplitude as describe in rodents (Lavrov et al., 2006). Overall, our results show the feasibility of the swine as an intermediary model to study EES evoked motor responses in SCI models and could be combined in the investigation of autonomic functions as showed recently by Guiho et al. (2017).

### The Amplitude of the EES Evoked Motor Responses Depends on Stimulating Electrode Proximity to Dorsal Root Entry Zone

In previous studies of healthy (Gerasimenko et al., 2006) and chronic SCI rats (Lavrov et al., 2006, 2008; Courtine et al., 2009; Nandra et al., 2011; Gad et al., 2013), epidural electrodes were placed on the midline of the spinal cord, typically, at L2 and/or S1, and different motor responses based on amplitude and waveform were observed during stimulation. These differences were never related with the electrode position in relation to the spinal cord dorsal structures. Our results show that EES delivered close to the dorsal rootlets entry zone provide the most robust motor responses (Figures 6, 7). In general, the amplitude of the motor evoked responses was higher with the electrode placed in proximity to the dorsal roots, compared to the responses recorded with the stimulating electrode in between the dorsal roots entry (Figures 6, 7). The latter was clearly evident with single electrode stimulation over the L1, L2 and L3 dorsal root entry zones, compared to EES at intermediate positions (L1-L2, L2-L3 and L3-L4). In fact, changes in EES evoked motor responses were similar in proximal and distal muscles with exception of the relative high amplitude in TA when EES was applied at L2-L3 (Figure 6A). Surprisingly, the influence of electrode placement in relation to the dorsal roots was even more critical than position of electrode in relation to different spinal segments (rostral vs. caudal electrode position). For example, maximal peak-to-peak amplitudes were observed when EES was delivered at L3 in most muscles recorded, but no significant differences were found when comparing EES at L1 or L2 (Figure 6A), where electrode was located ≈5.2 cm and ≈2.6 cm apart, respectively. Moreover, a dramatic decrease in amplitude was observed when comparing nearby electrode locations, for example, the distance between L3 and L3-L4 electrode positions was 1 cm approximately but the difference in amplitudes was up to 90% in proximal and 60% in distal muscles (Figure 6A). When the multi-contact rod array was placed on the midline of the spinal cord spanning L4-L6, maximum amplitudes (100%) were observed during stimulation at locations close to the dorsal roots entrance zones, for example at L6 for GLU, GAS and SOL, while EES applied at L4 produced maximal amplitude in RF. At the same time, BF and TA amplitudes were maximal when stimulating at L5-L6 (Figure 7A). Minimal amplitudes were observed in all muscles when EES was applied at L4-L5 (Figure 7A). Interestingly, increasing amplitudes from L4-L5 electrode position to L6 was observed in GLU, GAS and SOL muscles and similar pattern was observed in RF and TA muscles with maximal amplitudes at L5-L6. The proximity of dorsal roots particularly between L5 and L6 could in part explain our results. Location of stimulating electrodes proximal to the dorsal roots in L5-L6 could provide more specific responses as observed using the single electrode in L1, L2 and L3 (Figure 6). In summary, the electrode position in relation to the dorsal roots anatomy is a critical factor that produces a stronger impact on EES rather than shifting the electrode to rostral or caudal segments.

### Implications and Future Directions

Available computational models of EES effect are primarily based on rodent anatomical data, however, the anatomy of the dorsal roots and rootlets has not been considered as a part of these models. In some models angles of rostral and caudal roots from 0 to 45° in respect to the transverse plane were incorporated, (Struijk et al., 1993; Lempka et al., 2015), however without natural variation from segment to segment that makes knowledge about each segment anatomy critical for neuromodulation strategies. The anatomical description of the dorsal anatomy of the spinal cord provided here, could be used for development of computational simulations and contribute to future spinal cord neuromodulation studies, the effect of EES on bladder and bowel functions (for example, see Guiho et al., 2017), as well as long term therapeutic effect for SCI. Also, considering the advantages of the swine model over rodents in terms of size and similarities with humans in relation to spinal anatomy, the delivery of orientation-selective EES could be achieved taking into account the differences in neuroanatomy across different spinal cord segments (Figures 1, 2, Tables 1–4) and considering the importance of electrode position in relation to the dorsal roots (Figures 6, 7).

## Conclusion

In this study, evaluation of functional neuroanatomy of the swine spinal cord was performed based on EES evoked motor responses. Examination of anatomical spine and spinal cord landmarks showed significant differences from rostral to caudal lumbar segments and particularly between L1-L3 and L5-L6. Among the intersegmental landmarks, the intervertebral spinous process length and particularly at L2, could be used as an anatomical reference to establish a relationship between the spine and spinal cord. In this study, we show for the first time, that the amplitude of the EES evoked motor responses, particularly the MR (monosynaptic response), dramatically depends on the position of the stimulating electrode in relationship to the dorsal root entry zones. Taken together, these results provide a new understanding of the effect of EES and also establish a detailed anatomical picture of the functional neuroanatomy of the spinal cord in swine, which will help to reduce the gap in translation of spinal cord neuromodulation techniques from animal research to clinical studies.

## Author Contributions

CAC, AAM, PJG and IAL designed model framework. CAC, AAM, RI, JSC, BK and IAL conducted the data collection and electrophysiology experiments. CAC, AAM, RI and TP analyzed data. KHL and IAL approved the final version of the manuscript. All authors read and helped to improve the manuscript.

## Conflict of Interest Statement

The authors declare that the research was conducted in the absence of any commercial or financial relationships that could be construed as a potential conflict of interest.

## Footnotes

^1^www.geogebra.org
